# Single-layer six-band metasurface absorber for microwave applications

**DOI:** 10.1371/journal.pone.0353606

**Published:** 2026-08-10

**Authors:** Hattan Abutarboush, Syed Wahab Zarin, Aamir Rashid, Qasim Awais, Farooq A. Tahir

**Affiliations:** 1 Department of Electrical Engineering, College of Engineering, Taibah University, Madinah, Saudi Arabia; 2 School of Electrical Engineering and Computer Science, National University of Sciences and Technology (NUST), Islamabad, Pakistan; 3 Department of Electronics Engineering, University of Engineering and Technology, Taxila, Pakistan; 4 Department of Electronic Engineering, Fatima Jinnah Women University, Rawalpindi, Pakistan; 5 James Watt School of Engineering, University of Glasgow, Glasgow, United Kingdom; Guru Ghasidas University Department of Pure & Applied Physics, INDIA

## Abstract

A single-layer six-band metasurface absorber for microwave applications is presented, fabricated, and experimentally validated. The proposed unit cell is composed of concentric inner and outer ring resonators, with four symmetrically placed stubs on the outer ring at 90° intervals. The structure is printed on a 2.4 mm thick FR4 substrate with a loss tangent of 0.02 and backed by a continuous metallic ground plane. The novelty of the design lies in achieving six distinct absorption bands using a simple single-layer ring-based geometry, without requiring multilayer stacking or additional lumped components. The absorber operates over the frequency ranges of 2.278–2.317 GHz, 4.496–4.571 GHz, 10.44–10.59 GHz, 13.518–13.65 GHz, 14.66–14.82 GHz, and 17.77–18.70 GHz. A comparatively wide absorption bandwidth of 930 MHz is obtained in the highest-frequency band. The absorption magnitude remains above 90% across all six operating bands. The absorption mechanism is investigated through surface impedance matching, C4-symmetric current cancellation, and surface current distribution analysis, showing that the lower bands are mainly associated with magnetic resonances, while the higher bands originate from electric and hybrid resonant modes. The simulated and measured results show good agreement, confirming the effectiveness of the proposed metasurface absorber for multiband microwave absorption applications.

## 1. Introduction

Metasurfaces, the two-dimensional analogue of metamaterials, are artificially engineered periodic structures that can exhibit electromagnetic properties not commonly available in natural materials. During the last two decades, metasurfaces have attracted considerable attention due to their potential applications in polarization control, negative refraction, cloaking, super-lensing, radar cross-section reduction, and electromagnetic wave absorption [1–6]. Among these applications, metasurface-based electromagnetic absorbers are particularly important for microwave systems, stealth technology, electromagnetic shielding, sensing, and radar cross-section reduction.

A major challenge in metasurface absorber design is to achieve multiple absorption bands, high absorption efficiency, compact geometry, and low structural complexity at the same time. Conventional and multilayer absorber technologies can provide useful absorption performance, but they often involve increased thickness, expensive material platforms, multilayer stacking, alignment requirements, or complex fabrication [5–11]. Therefore, reducing structural complexity while retaining efficient multiband absorption remains an important design objective.

There is therefore a strong demand for simple, low-profile, single-layer metasurface absorbers that can provide multiband absorption while remaining easy to fabricate. Several single-layer absorbers have been reported in the literature, but many of them provide only one, two, or three absorption bands, or require more complex resonator shapes to achieve multiband performance [12,13,14–24]. Some recent single-layer absorbers have also been reported for microwave and 5G-related applications [25–27]. Although these studies demonstrate important progress, the simultaneous realization of six absorption bands using a compact and simple single-layer geometry on a low-cost FR4 substrate remains a challenging design problem.

Another important issue in the literature is the distinction between true absorption and polarization conversion. Some broadband structures reported as absorbers are based on anisotropic unit cells that convert the incident field into the orthogonal polarization rather than fully dissipating the incident electromagnetic energy [28–30]. For a metasurface absorber backed by a metallic ground plane, the transmission is suppressed, and high absorption requires the reflected co- and cross-polarized components to be minimized. Therefore, symmetric unit-cell design, impedance matching, and surface-current cancellation are important for achieving effective absorption.

In this study, a single-layer six-band metasurface absorber for microwave applications is proposed, fabricated, and experimentally validated. The unit cell consists of concentric inner and outer rings, with four stubs symmetrically loaded on the outer ring at 90° intervals. The structure is fabricated on an inexpensive FR4 substrate with a thickness of 2.4 mm and backed by a continuous metallic ground plane. The central novelty of this work is that six distinct absorption bands are achieved using a simple single-layer ring-based geometry, without using multilayer stacking or additional lumped loading. Compared with many reported single-layer absorbers, which commonly provide fewer operating bands or require more complicated geometries, the proposed design offers a compact and fabrication-friendly route to multiband microwave absorption.

The proposed absorber operates over six frequency ranges: 2.278–2.317 GHz, 4.496–4.571 GHz, 10.44–10.59 GHz, 13.518–13.65 GHz, 14.66–14.82 GHz, and 17.77–18.70 GHz. The absorption remains above 90% across all six operating bands, with a comparatively wide 930 MHz absorption bandwidth in the highest-frequency band. The absorption mechanism is analysed using surface impedance matching, C4-symmetric current cancellation, and surface current distribution analysis. The lower-frequency absorption bands are mainly associated with magnetic resonances, while the higher-frequency bands arise from electric and hybrid resonant modes supported by the coupled ring structure. The simulated and measured results show good agreement, confirming the effectiveness of the proposed single-layer six-band metasurface absorber.

## 2. Results and discussion

**Fig 1:**
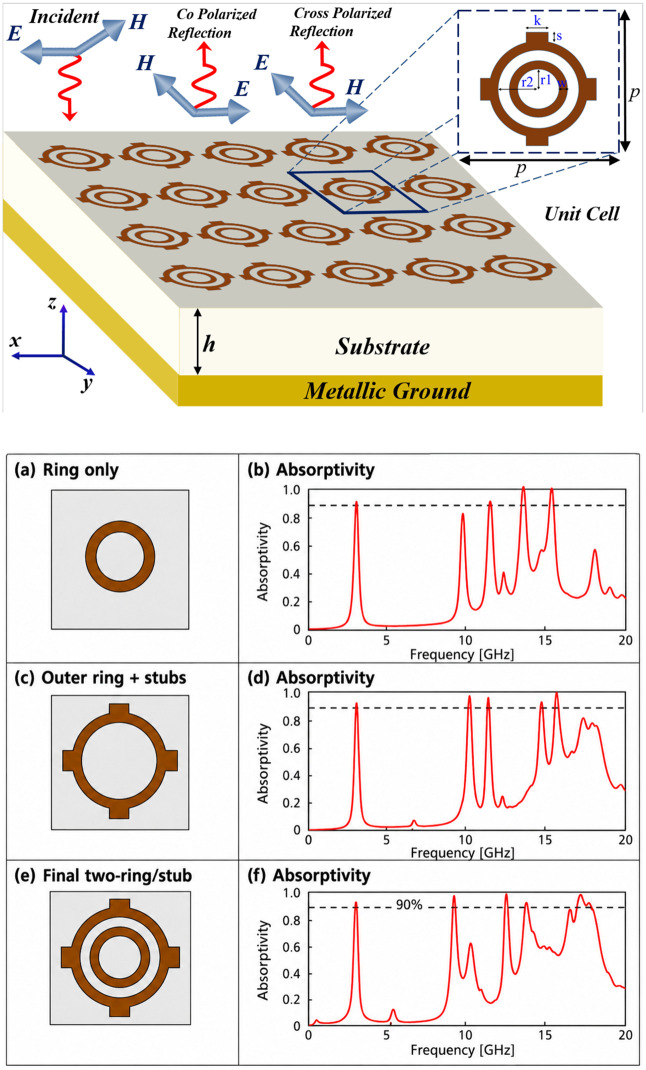
(a) 3D schematic diagram of the proposed absorber (b) Design evolution of the proposed metasurface absorber: (a) ring-only unit cell and (b) corresponding absorptivity; (c) outer ring with four stubs and (d) corresponding absorptivity; (e) final two-ring/stub unit cell and (f) corresponding absorptivity.


A three-dimensional schematic of the proposed metasurface absorber is shown in Fig 1(a) The initial resonator dimensions were estimated using the basic half-wavelength design guideline for a single-layer metasurface absorber,

$$\begin{array}{c} L=n\;.\;\frac{\lambda_{eff}}{\text{2}},\text{ n}=\text{1},\text{2},\text{3},\ldots \end{array}$$

where L is the effective resonant length and λeff is the effective wavelength in the dielectric medium [11]. The final dimensions were then obtained through full-wave parametric optimization. The optimized geometrical parameters are p = 30 mm, s = 1.6 mm, w = 2 mm, k = 4 mm, r1 = 5 mm, r2 = 10 mm, and h = 2.4 mm.

The metasurface consists of a two-dimensional periodic array of identical unit cells. Each unit cell is composed of two concentric metallic rings, where the outer ring is additionally loaded with four identical stubs placed symmetrically at 90° intervals. This compact ring-stub configuration enables multiple resonant current paths within a single unit cell, which is essential for achieving six absorption bands without increasing the structural profile. The patterned metallic layer is printed on an FR4 substrate with a loss tangent of tanδ = 0.02, and the structure is backed by a continuous metallic ground plane to suppress transmission. All metallic layers are made of copper with a thickness of 0.035 mm. The unit-cell footprint is 30 × 30 mm^2^, while the total absorber thickness is 2.4 mm.

The subject metasurface is designed using CST Microwave Studio with periodic boundary conditions (PBCs) on the four sides. The Floquet port is used to simulate the incident plane wave in the periodic environment. A step-by-step design evolution was also carried out to justify the final unit-cell configuration, as shown in Fig 1(b). The first stage used a single C4-symmetric ring resonator, which produced several narrow absorption peaks but did not provide the required six-band response. In the second stage, four equally spaced stubs were added to the outer ring to increase the effective current path and introduce additional resonant modes. In the final stage, the inner ring was combined with the stub-loaded outer ring, allowing independent and coupled resonant current paths to be excited within the same compact unit cell.

The corresponding absorptivity plots show that the ring-only and stub-loaded outer-ring configurations generate multiband absorption with comparatively narrow and incomplete responses. In contrast, the final two-ring/stub configuration produces six absorption bands across the S-, C-, X-, Ku-, and K-band regions, including the comparatively broad high-frequency absorption band. This design evolution supports the use of the concentric-ring and stub-loaded geometry for achieving compact single-layer six-band absorption.

Absorptivity A (ω) is evaluated from the reflection R(ω) and transmission T(ω) coefficients [13], as given in Eq. (1).


$$\begin{array}{c} \;A\;(\omega )=\text{1}-{\left|R(\omega )\right|}^{\text{2}}-{\left|T(\omega )\right|}^{\text{2}} \end{array}$$ (1)

Where ${\left|R(\omega )\right|}^{\text{2}}=\;|{\text{R}}_{\text{xx}}{|}^{\text{2}}+|{{\text{R}}_{\text{yx}}|}^{\text{2}}\;and\;{\left|T(\omega )\right|}^{\text{2}}=|{{\text{T}}_{\text{xx}}|}^{\text{2}}+|{{\text{T}}_{\text{yx}}|}^{\text{2}}$ represent the total reflected and transmitted powers, respectively. The ${\text{R}}_{\text{xx}}$ and ${\text{R}}_{\text{yx}}$ are co- and cross-polarization reflection coefficients, respectively, when the incident electric field is along x-axis and vice versa. The value of the total transmitted power ${\left|T(\omega )\right|}^{\text{2}}$ is zero as the metasurface is backed by a metallic ground plane. The Fresnel expression for the reflected power$\;{\left|R(\omega )\right|}^{\text{2}}$ is given below, wherein, ${\epsilon \;}_{r}(\omega )$ and $\mu_{r}(\omega )$ are respectively the frequency-dependent relative permittivity and permeability of the metasurface.


$$\begin{array}{c} \;{\left|R(\omega )\right|}^{\text{2}}={\left|\frac{Z_{A}(\omega )-Z_{O}}{Z_{A}(\omega )+Z_{O}}\right|}^{\text{2}}={\left|\frac{\sqrt{\mu_{r}(\omega )}-\sqrt{{\epsilon \;}_{r}(\omega )}}{\mu_{r}(\omega )+\sqrt{{\epsilon \;}_{r}(\omega )}}\right|}^{\text{2}} \end{array}$$ (2)

Where ${\text{Z}}_{\text{0}}(=\text{377}\Omega )\;$is free space impedance of the wave and $Z_{A}(\omega )\;(=\sqrt{\frac{\mu_{A}(\omega )}{\varepsilon_{A}(\omega )}}\times \text{377})$ is surface impedance of the absorber [11]. It can be seen from Eq. (2), that maximum absorption is achieved when $Z_{A}(\omega )$ approaches the value of free space impedance ${\text{ Z}}_{\text{0}}$ and it happens when $\;{\epsilon \;}_{A}(\omega )\approx \mu_{A}(\omega ).$ So, the absorption condition of $\sqrt{\frac{\mu_{\text{A}}(\omega )}{\epsilon_{\text{A}}(\omega )}}=\text{1}$ can be achieved through a judicious design of the unit cell.

**Fig 2:**
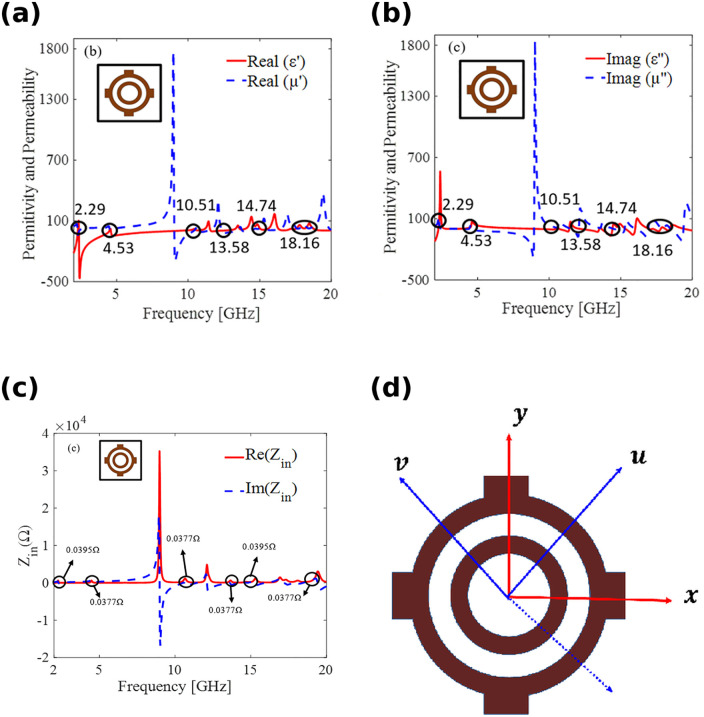
(a) Real parts of the permittivity and permeability (b) Imaginary parts of the permittivity and permeability (c) The effective surface impedance of the proposed absorber, (d) Depiction of C4 symmetry of the unit cell.

As shown in Fig 2(a) and 2(b), the real as well as imaginary parts of the complex permittivity and permeability become approximately equal at the center frequency of all the six-frequency bands so the metasurface impedance $Z_{A}(\omega )\;$ is well matched with the free space impedance ${\text{Z}}_{\text{0}}$=377Ω as shown in Fig 2(c) allowing the EM wave incident on the metasurface with almost negligible reflection.

Another aspect critical to the absorbing surface is its C4 (four corner) symmetrical geometrical structure. The C4 symmetry means that the mirror image of the surface along both x- and y-axes must overlap with each other. The C4 symmetrical structure is necessary to reduce any reflection in the form of cross component from the absorbing surface due to null vector sum of surface currents. The subject unit cell, as depicted in Fig 2(d) has C4 symmetry along both x- and y-axis.

The overall surface currents are therefore canceled out on the metallic metasurface layer and none of the electric field components are reflected as $E_{rx}$, $E_{ry}$.

**Fig 3:**
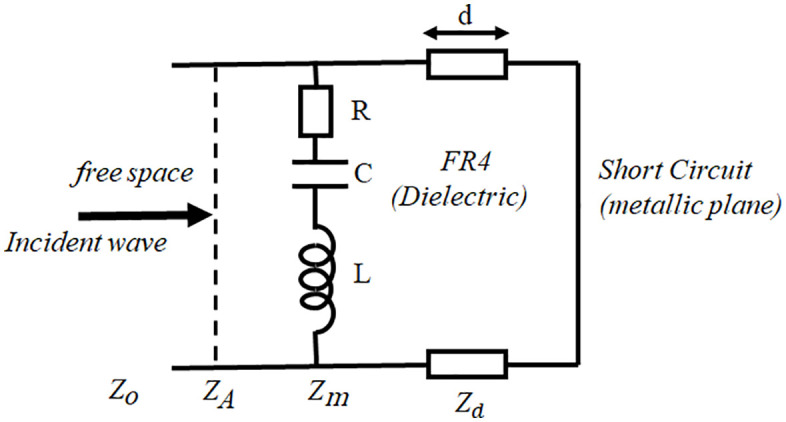
A general Circuit Model representation of the Metasurface-based absorber.

The proposed absorber can be described through a general equivalent circuit as shown in Fig 3 [31,32]. The upper metallic layer can be represented by an equivalent RLC circuit. The metallic plane on the back is modeled as a short circuit, and dielectric layer is considered as a transmission line.

The impedance of the periodic metasurface and the dielectric layer can be written as,


$$\begin{array}{c} Z_{m}=R+j\omega L+\frac{\text{1}}{j\omega C} \end{array}$$ (3)

$$\begin{array}{c} Z_{d}=j{Z_{c}}^{TE,TM}/\text{tan}(\beta h) \end{array}$$ (4)

$Where\;{Z_{c}}^{TE}=\frac{\left(\omega {\mu_{r}\mu }_{\text{0}}\right)}{\beta }and\;{Z_{c}}^{TM}=\beta /(\omega {\varepsilon_{r}\varepsilon }_{\text{0}})$, are the characteristic impedances of the dielectric layer short-circuited with metallic ground plane and h is the thickness of the dielectric layer. The propagation constant $\;\beta =\frac{\omega }{c_{\text{0}}}\sqrt{\varepsilon_{r}\mu_{r}-{(\text{sin}\theta )}^{\text{2}}}$ is taken along the normal with θ is the incident angle and $c_{\text{0}}$ is the wave velocity in vacuum. The surface impedance $Z_{A}\;$of the absorber is equal to the parallel sum of the impedances $\;Z_{m}$ and $Z_{d}$ and can be written as:


$$\begin{array}{c} {\text{ Z}}_{\text{A}}=\frac{{\text{Z}}_{\text{m}}{\text{Z}}_{\text{d}}}{{\text{Z}}_{\text{m}+}{\text{Z}}_{\text{d}}} \end{array}$$ (5)

The reflection coefficient is then given by:


$$\begin{array}{c} \;\left|R(\omega )\right|={\left|\frac{Z_{A}(\omega )-Z_{O}}{Z_{A}(\omega )+Z_{O}}\right|}^{\text{2}} \end{array}$$

Where for TE waves, for TM waves [31,32]. $Z_{\text{0}}=\frac{\sqrt{\frac{\mu_{\text{0}}}{\varepsilon_{\text{0}}}}}{\text{cos}\theta }{\;Z}_{\text{0}}=\sqrt{\frac{\mu_{\text{0}}}{\varepsilon_{\text{0}}}}\text{cos}\theta$

**Fig 4:**
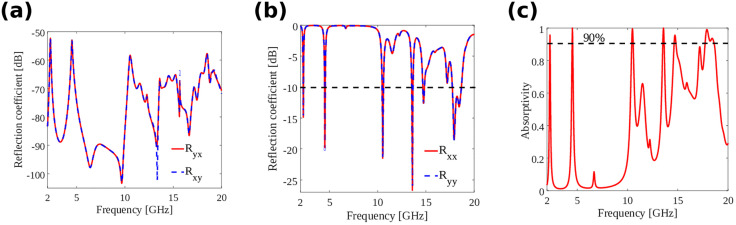
(a) Co-pol reflection coefficient (b) Cross-pol reflection coefficient (c) Absorptivity (c) Linear absorption.

The reflection coefficients for both co- and cross-polarizations are shown in Fig 4. The values of both co- and cross-polarized reflection coefficients for normal incidence are well below −10dB at six distinct frequency bands, which are 2.278–2.3171 GHz, 4.496–4.571 GHz, 10.44–10.59 GHz, 13.518–13.65 GHz, 14.66 to 14.82 GHz and 17.77–18.70 GHz. Comparatively, broad absorption bandwidth of 930 MHz is achieved at the last frequency band. As depicted in Fig 4(a) and 4(b), the response of the metasurface is same for both x and y polarized incident waves due to pure mirror symmetry. As shown in Fig 4(c), the absorption of the proposed metasurface is above 90% rather it is approaching unity for all six-frequency bands.

The polarization response is therefore governed by the four-fold symmetry of the unit cell. Since the geometry is symmetric along the x- and y-axes, the induced currents follow equivalent paths for orthogonal incident polarizations, giving nearly identical Rxx and Ryy responses. The cross-polarized reflected component remains very small because the orthogonal current components are balanced by the C4-symmetric layout.

**Fig 5:**
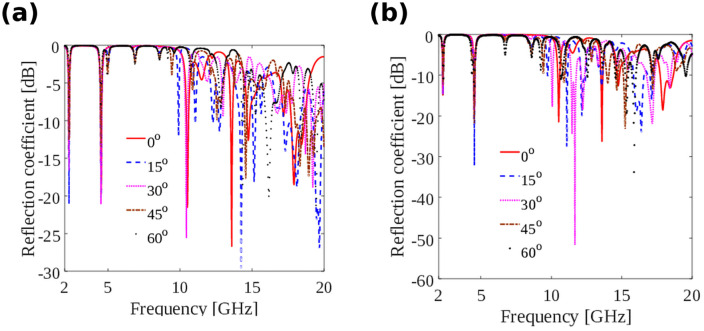
(a) Angular stability for x- Polarization (b) Angular stability for y- Polarization.

Angular stability is a key requirement to enhance the practicality of any absorbing surface. Fig 5(a) and 5(b) show the angular stability of co-polarized reflection coefficients for both x-and y-polarized incident waves. The absorber achieved angular stability up to 60° at the two lower frequency bands for both x and y polarizations. The other four frequency bands are angularly stable up to 30°.

The stronger angular stability at the two lower-frequency bands can be attributed to the longer resonant current paths associated with the inner and outer rings and the smaller electrical size of the unit cell relative to the wavelength at these bands. Under oblique incidence, the phase variation across the unit cell is therefore less pronounced, so the resonant response is preserved up to 60°. In contrast, the four higher-frequency bands arise from higher-order electric and hybrid modes; these modes are more sensitive to incidence angle because the unit cell becomes electrically larger and the field distribution varies more strongly across the resonator. Consequently, these bands maintain stable absorption up to approximately 30°.

**Fig 6:**
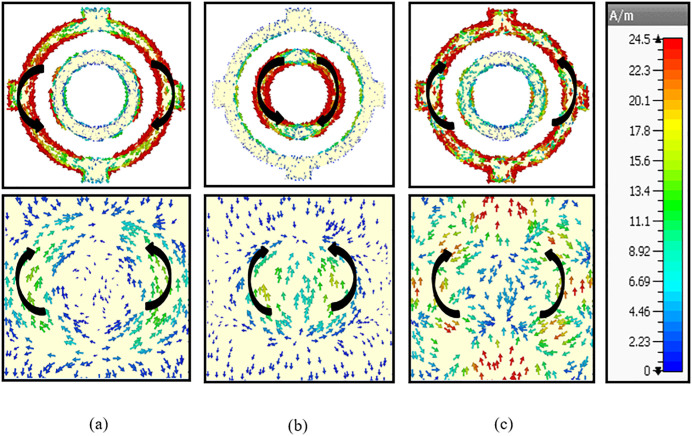
Surface currents distribution of upper metallic layer and ground (a) Magnetic resonance at 2.5GHz (b) Magnetic resonance 4.5GHz (c) Electric resonance at 10.5GHz.

**Fig 7:**
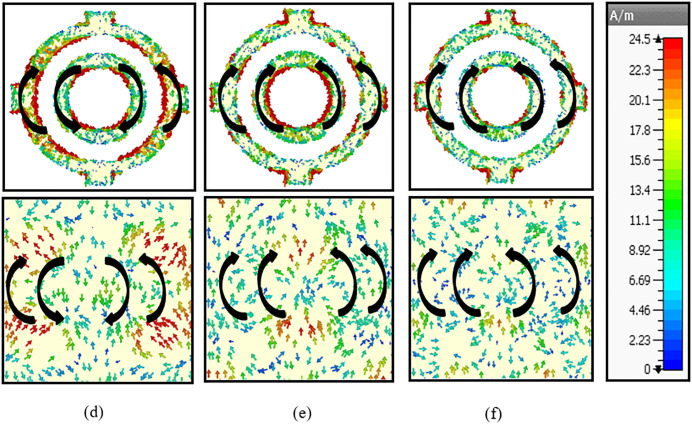
Surface currents distribution of upper metallic layer and ground Electric resonances at (a) 13.5GHz (b) 14.5GHz (c) 18GHz.

To further examine the absorption mechanism of the proposed metasurface, the surface current distributions are analysed, as shown in Figs 6 and 7. The current distribution on the upper metallic layer is mainly confined to the two concentric rings, where different resonant paths are excited at different operating frequencies. At the lower two absorption bands, magnetic resonances are formed because the surface currents on the top metallic layer flow in the opposite direction to those on the ground plane. In contrast, the remaining four absorption bands are mainly associated with electric resonances, where the currents on the upper metallic layer and the ground plane flow in the same direction.

As shown in Fig 6(a) and 6(b), the lowest absorption band centred at 2.5 GHz is primarily generated by the outer ring, while the second absorption band centred at 4.5 GHz is mainly associated with the inner ring. The third absorption band centred at 10.5 GHz, shown in Fig 6(c), corresponds to a higher-order electric resonant mode of the outer ring. These current distributions confirm that the compact ring-based unit cell supports multiple resonant modes, which collectively contribute to the six-band absorption response of the proposed metasurface.

Similarly, the remaining three absorption bands centred at 13.5 GHz, 14.5 GHz, and 18 GHz are produced by hybrid resonant modes arising from the electromagnetic coupling between the inner and outer rings, as shown in Fig 7(a)–7(c). These hybrid modes confirm that the proposed compact ring-based geometry supports several independent and coupled resonant paths within the same unit cell.

The C4-symmetric geometry further contributes to the absorption behaviour by suppressing unwanted cross-polarized reflection. Due to the symmetry of the structure, the induced surface currents are balanced in orthogonal directions, which reduces the reflected co- and cross-polarized field components. As a result, most of the incident electromagnetic energy is dissipated within the lossy FR4 substrate, leading to high absorption at the resonant frequencies.

## 3. Experimental validation

**Fig 8:**
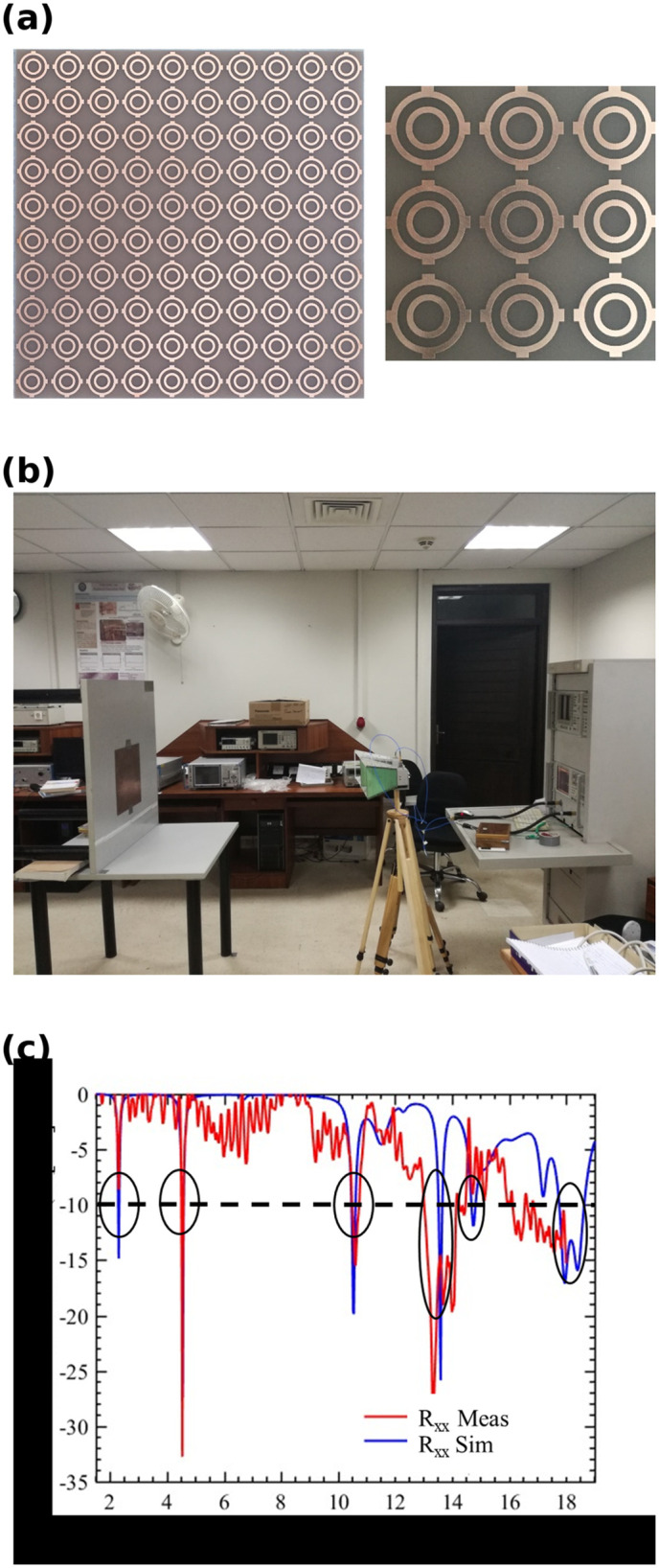
(a) Fabricated Prototype (b) Measurement setup (c) Simulated and measured co- polarized reflection coefficients.

To experimentally validate the proposed absorber design, a prototype was fabricated on a 304.8 × 304.8 mm^2^ FR4 substrate backed by a continuous copper ground plane, as shown in Fig 8(a). The prototype was fabricated using a standard printed circuit board (PCB) process.

The free-space reflection measurements were carried out in the laboratory using two double-ridged broadband horn antennas connected to an Agilent E8362B vector network analyzer (VNA), as shown in Fig 8(b). The fabricated metasurface sample was placed in front of the transmitting and receiving horn antennas approximately at the far-field distance, and a free-space calibration procedure was performed before recording the reflection response. The experimental arrangement was used to measure the reflected field components from the fabricated absorber.

Although the present work is validated using a free-space reflection measurement setup, reverberation chambers can also provide an alternative environment for evaluating absorber performance under statistically uniform and multipath field conditions, where the working volume and field uniformity are important for reliable measurements [33].

For the measurement of the co-polarized reflection coefficient Rxx, both horn antennas were aligned in the horizontal orientation corresponding to x-polarization. The simulated and measured Rxx responses are shown in Fig 8(c). Similarly, for the measurement of Ryy, both antennas were aligned in the vertical orientation corresponding to y-polarization. Due to the four-fold symmetry of the proposed unit cell, the cross-polarized reflection coefficient Ryx remains very small and is therefore not shown separately.

The measured results show good agreement with the simulated response, confirming the validity of the proposed absorber design. The small differences between simulated and measured results can be attributed to the finite size of the fabricated prototype, fabrication tolerances, material parameter variations in the FR4 substrate, and practical limitations of the free-space measurement setup. Table 1 presents a comparison between the proposed design and previously reported single-layer metasurface absorbers. The comparison highlights that the proposed absorber achieves six absorption bands using a compact single-layer geometry on an inexpensive FR4 substrate, while maintaining high absorption efficiency and a comparatively wide absorption bandwidth in the highest-frequency band.

**Table 1:** Comparison table.

Ref	Unit cell size (mm3)	Shape of the structure	Dielectric material used	Absorption peak freq (GHz)	Absorptivity (%)	Polarization insensitive Nature
14	14 × 14 × 1.6	Circular slot type	FR4	2.9, 4.18	95, 95.45	yes
15	10 × 10 × 1.6	Circular ring type	FR4	9.25, 5.57, 7.97, 13.44	97.2, 96.87, 95.99, 98.28	yes
16	13.8 × 13.8 × 1.6	Circular ring with an inner Jerusalem cross	FR4	4.4, 6.05, 13.9	93.13, 96.41, 96.36	yes
17	28.2 × 28.2 × 1.6	Square type ring with a Jerusalem cross	FR4	4.20, 7.00, 7.4	91, 97.9, 98.5	yes
18	12 × 12 × 1.6	Dumbbell	FR4	3.26, 11.6, 17.13	93.8, 96.74, 99.95	yes
19	35 × 35 × 1.6	Square and circular type SRR	Rogers	6.46, 7.68	97.1, 91.2	no
20	11.5 × 11.5 × 1.6	Jerusalem cross	PET	6.1, 9.2, 11.6	95, 96, 97	yes
21	10 × 10 × 1.6	Rhombus	FR4_Epoxy	5.86, 6.57, 8.94	86.23, 92.92, 82.07	yes
22	10 × 10 × 0.1	Cross	Polyimide	11.4, 19.2	97.5, 96.5	yes
23	42 × 42 × 0.1	Non uniform size of different polygon shapes	Jeans	9.98, 10.43, 11.3	95.1, 97.3, 99.89	yes
This work	30 × 30 × 2.4	Double ring shapes	FR4	2.29,4.53,10.51,13.5,14.74,18.19	98, 99, 99, 99, 98, 97	yes

## 4. Conclusion

A single-layer six-band metasurface absorber for microwave applications has been presented, fabricated, and experimentally validated. The proposed absorber operates over six distinct frequency bands: 2.278–2.317 GHz, 4.496–4.571 GHz, 10.44–10.59 GHz, 13.518–13.65 GHz, 14.66–14.82 GHz, and 17.77–18.70 GHz. The absorption magnitude remains above 90% across all six operating bands, with a comparatively wide absorption bandwidth of 930 MHz achieved in the highest-frequency band.

The proposed design demonstrates that multiple absorption bands can be realised using a compact single-layer ring-based metasurface printed on a low-cost FR4 substrate. The absorption mechanism has been explained through impedance matching, C4-symmetric geometry, and surface current distribution analysis. The lower-frequency bands are mainly associated with magnetic resonances, while the higher-frequency bands are produced by electric and hybrid resonant modes arising from the coupled inner and outer rings.

The simulated and measured results show good agreement, confirming the validity of the proposed absorber. The combination of six-band operation, simple single-layer geometry, compact unit-cell structure, low-cost substrate, and experimentally verified absorption performance makes the proposed metasurface absorber a promising candidate for multiband microwave absorption applications.
